# PyCellBase, an efficient python package for easy retrieval of biological data from heterogeneous sources

**DOI:** 10.1186/s12859-019-2726-4

**Published:** 2019-03-28

**Authors:** Daniel Perez-Gil, Francisco J. Lopez, Joaquin Dopazo, Pablo Marin-Garcia, Augusto Rendon, Ignacio Medina

**Affiliations:** 1 Genomics England, London, UK; 2 Clinical Bioinformatics Area, Fundacion Progreso y Salud, Seville, Spain; 3 Functional Genomics Node, INB-ELIXIR-es, FPS, Hospital Virgen del Rocío, Seville, Spain; 4 Department of Bioinformatics, Universidad Católica de Valencia, Valencia, Spain; 5 Department of Bioinformatics, Institute for Integrative Systems Biology, Valencia, Spain; 6 Department of Haematology, University of Cambridge, Cambridge, UK; 7 HPC Service, UIS, University of Cambridge, Cambridge, UK

**Keywords:** Annotation, Variant, CellBase, Database, Repository, RESTful, Python

## Abstract

**Background:**

Biological databases and repositories are incrementing in diversity and complexity over the years. This rapid expansion of current and new sources of biological knowledge raises serious problems of data accessibility and integration. To handle the growing necessity of unification, CellBase was created as an integrative solution. CellBase provides a centralized NoSQL database containing biological information from different and heterogeneous sources. Access to this information is done through a RESTful web service API, which provides an efficient interface to the data.

**Results:**

In this work we present PyCellBase, a Python package that provides programmatic access to the rich RESTful web service API offered by CellBase. This package offers a fast and user-friendly access to biological information without the need of installing any local database. In addition, a series of command-line tools are provided to perform common bioinformatic tasks, such as variant annotation. CellBase data is always available by a high-availability cluster and queries have been tuned to ensure a real-time performance.

**Conclusion:**

PyCellBase is an open-source Python package that provides an efficient access to heterogeneous biological information. It allows to perform tasks that require a comprehensive set of knowledge resources, as for example variant annotation. Queries can be easily fine-tuned to retrieve the desired information of particular biological features. PyCellBase offers the convenience of an object-oriented scripting language and provides the ability to integrate the obtained results into other Python applications and pipelines.

**Electronic supplementary material:**

The online version of this article (10.1186/s12859-019-2726-4) contains supplementary material, which is available to authorized users.

## Background

During the past years, the increase in scientific knowledge due to the massive data production from high-throughput technologies have caused an unprecedented growth in the number and size of databases storing relevant biological data [1]. However, these annotations are fragmented among many resources that range greatly in terms of capacity, scope and organization (e.g., Ensembl [2], UniProt [3], and Reactome [4]). As size, diversity and complexity of these biological repositories expands, serious functionality problems arise in terms of access through Internet and storage in local disks [5]. In addition, most of relevant data is stored in different repositories or databases and different standards and identifiers are used [6]. In particular, annotations of human genes and variants are broadly used both in research and in clinical practice but highly spread across many resources.

Tools for knowledge integration enable more efficient analysis of genome-scale data sets and discovery of relationships between biological entities [7, 8]. CellBase arose as a solution to the growing necessity of integration by easing the access to biological data [9]. CellBase provides a centralized NoSQL database containing biological information and a RESTful web service API to query these data. Users can access to multiple resources for different features such as genes, transcripts, proteins, variations or clinical data. CellBase has been used in applications for variant prioritization [10] and it is used for variant annotation in the 100,000 Genomes Project [11].

To query data from CellBase, users have to access its RESTful web services. Therefore, they are required to know how the URL queries are constructed and be capable of managing the server responses. Such operations can turn out to be tedious, repetitive and error-prone.

To address these issues, we have developed PyCellBase, an efficient Python package that provides programmatic access to CellBase biological information. Python was chosen as programming language because it is open-source, runs on all major operating systems [12] and has become very popular for scientific programming [13]. PyCellBase consumes the RESTful web services to acquire data from CellBase, providing a simple and fast access to the database. A series of clients and methods have been implemented to retrieve specific resources from the main features stored in CellBase. PyCellBase provides the advantages of an object-oriented scripting language and allows the integration of queries to the RESTful web service into Python scripts with no need to know how the service works. In addition, several tools are also provided to facilitate recurrent bioinformatics tasks, such as variant annotation.

## Implementation

### REST client library

**Fig. 1:**
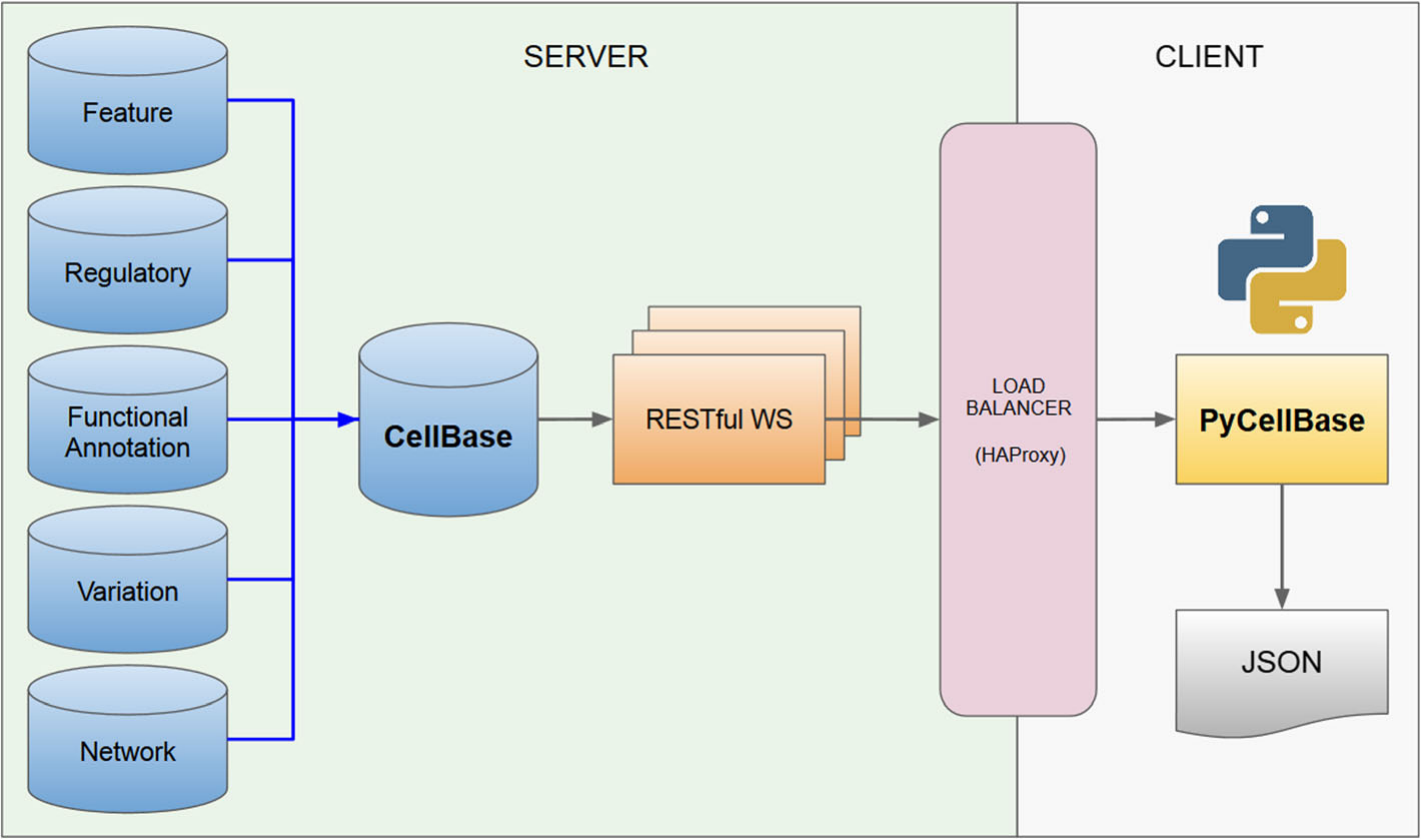
PyCellBase and CellBase architecture overview. Data from different biological databases and sources are integrated into CellBase, which implements a set of RESTful web services that query a centralized database containing the most relevant biological data sources. These RESTful web services are used by PyCellBase to query and fetch desired data in JSON format for further analysis and integration with other applications

PyCellBase uses the comprehensive RESTful web service API that has been implemented for the CellBase database (Fig. 1) and it can access any of the several public servers. Setting up this package is quick and simple, without the need of any local database installation. Only a minimal user configuration is required.

CellBase integrates different data types from different sources and its RESTful web services are organised in different categories depending on the type of information. To provide a clear way to obtain these data, PyCellBase implements a main client (*CellBaseClient*) that creates different resource-specific clients for each category of biological data (Table 1) such as *GeneClient* to query genes or *VariantClient* to annotate genomic variants.

**Table 1:** PyCellBase resource-specific clients and available data. The different clients that are currently implemented for each biological data type are shown along with the main data that can be obtained from each one of them

Client	Data
Gene	Clinical data, protein-protein interactions, biotype, TFBSs, expression, transcripts, protein, variants
Transcript	Sequence, function prediction, XREFs, biotype, genes, proteins, exons
Protein	Amino acid sequence, substitution scores, organism, genes, protein features, evidence, bibliography
Variation	Variants, biological impact, consequence types
Xref	Associated IDs from multiple databases (ENSEMBL, UCSC, UniProt, RefSeq, GO, Reactome, PDB, OMIM, RNAcentral, miRBase...)
Genomic region	Sequence, clinical variants, conservation scores, regulatory elements, repeat sequences, genes, transcripts, variants
Variant	Consequence types, genes, transcripts, proteins, population frequencies, HGVS, clinical significance, functional score
Genome sequence	Cytobands
Clinical	ClinVar, COSMIC, inheritance, evidence, pathogenicity, diseases
TFBS	Histones, open chromatin, polymerases, transcription factors

Additional clients have been designed to provide metadata about the available biological information and the RESTful web services. These additional clients can be used for example to retrieve information about the available data categories, species or database versioning.

Every resource-specific client provides a set of methods to fetch the desired information. Optional filters and extra options can be added to the query to narrow searches and reduce the amount of obtained information. To help the user select the appropriate parameters, every client provides a help method to list all the methods and filters that offers.

PyCellBase can retrieve information from all the 27 different species supported by CellBase, covering all major groups (metazoa, fungi, plantae, protista, bacteria and virus) with support for multiple genome assemblies. For example, for *Homo sapiens*, both GRCh37 and GRC38 are supported. In addition, most common ID formats from many independent bioinformatics databases are accepted when searching for biological data.

Configuration parameters such as the list of available RESTful web services hosts, API version and species can be customised. For this reason PyCellBase implements a client that manages the configuration (*ConfigClient*). A custom configuration can be stored in a file (JSON or YAML) or Python dictionary.

This package makes use of multithreading to improve performance when the number of queries exceed a specific limit. It also uses connection pooling and persists certain parameters across all connections, resulting in a significant performance increase.

PyCellBase returns results in Javascript Object Notation (JSON), which is a format that has been widely adopted in RESTful APIs [14]. JSON is designed to be a lightweight, language-independent data interchange format that is easy for humans to read and write, and for computers to generate and parse. This output format allows to seamlessly integrate results in other Python applications and pipelines.

### Command-line tools

In addition to PyCellBase core functionality, a command-line interface, called *cbtools.py*, has been implemented with several tools to ease and speed up frequently performed tasks in bioinformatics. These tools make use of the REST client library and offer a further output processing to facilitate its analysis.

#### ID converter

The heterogeneous and redundant nature of the different types of biological identifiers limits data analysis across different bioinformatics resources [7]. For this reason, the conversion of identifiers is one of the initial steps in many workflows related to genomic data analysis. To simplify this task, PyCellBase implements a gene ID converter (*cbtools.py xref*). This tool annotates genomic features with all their associated IDs, making use of 74 different sources for human, including most common databases such as Ensembl, NCBI, RefSeq, Reactome, OMIM, PDB, miRBase or UniProt among others. In addition, it supports heterogeneous input files with IDs from different sources.

#### HGVS calculator

The HGVS nomenclature recommendations for the description of sequence variants as originally proposed by the Human Genome Variation Society has gradually been accepted as the international standard for variant description [15]. Due to the wide use of this nomenclature for variants, mutations and polymorphisms in human health and diagnostics, the assignation of the specific HGVS name to a sequence variant is essential within research publications and clinical settings. For this reason, PyCellBase implements a tool that annotates variants with their associated HGVS names (*cbtools.py hgvs*). Given a file with multiple variants (in the format “*chromosome:position:reference:alternate*”), this tool returns all the associated HGVS names for many different types of reference sequence (non-coding, RNA, mitochondrial, genomic, protein, cDNA).

#### VCF annotator

Comprehensive variant annotations provide context that is crucial to variant interpretation for clinical diagnosis, medical databases and personalized medicine. Integration of genome annotations is critical to the identification of genetic variants that are relevant to studies of disease or other traits. PyCellBase takes advantage of the available integrated data that can access to implement a fast and rich variant annotator (*cbtools.py annotate*). This tool takes a VCF file as input and returns it with its variants annotated with a broad range of information such as consequence types, population frequencies, overlapping sequence repeats, cytobands, gene expression, conservation scores, clinical significance (ClinVar, COSMIC, diseases and drugs), functional scores and more. This VCF annotator is capable of annotating remotely an average of more than 500 variants per second with all the available data, using 1 processor in an ordinary laptop computer.

## Results

### Minimal example

PyCellBase implementation is focused on providing an efficient interface between the user and the data. For that reason it stands out for its simplicity and its ease of use.

For example, getting the annotation for a specific variant using the CellBase RESTful web service API requires to know how the URL query is constructed. In addition, configuration parameters have to be specified every time a query is done, which makes this system error-prone when the number of queries increase significantly:

- https://bioinfo.hpc.cam.ac.uk/cellbase/webservices/rest/v4/hsapiens/genomic/variant/17%3A430457%3AG%3AA/annotation?assembly=grch37

However, PyCellBase manages the configuration, the URL construction and handles the response. Therefore, the query above can be achieved in three simple steps:

1. Importing the PyCellBase module and initializing the main client.
  >>> from pycellbase.cbclient import CellBaseClient >>> cbc = CellBaseClient()
2. Creating the resource-specific client (variant client) for the query.
  >>>var_client = cbc.get_variant_client()
3. Obtaining annotation for the variant chr17:430457:G > A.
  >>> var_annot = var_client.get_annotation(‘17:430457:G:A’)

This manuscript is accompanied by a Jupyter Notebook [16] that provides a more exhaustive example [see Additional file 1].

### Usage

PyCellBase is used in several projects. It is worth noting its importance in the 100,000 Genomes Project (Genomics England). The objective of this project is to sequence 100,000 genomes of rare diseases and common cancers from the National Health System (NHS) patients and their families. This task requires access to a reliable source of information that could provide data in an efficient and easy way. For this reason, PyCellBase has been used as an annotation tool in variant prioritization and variant curation pipelines. The use of PyCellBase in such important large scale project proves the usefulness of this package and its straightforward usability.

### Development

PyCellBase development is synchronised with CellBase to ensure that the latest data and web services are always available. PyCellBase follows the Semantic Versioning rules [17]. Major and minor updates in the client are released along with CellBase updates, while fixes are released as necessary.

PyCellBase is a collaborative project that goes under continuous improvements and updates. In the near future, new features will be implemented, such as gRPC (gRPC, Remote Procedure Calls) support, common bioinformatics workflows and new resource-specific clients for new biological information such as biological networks.

## Conclusion

The biomedical research community has seen a proliferation of databases and web services in the past years. These services have become a primary source of information in the bioinformatics area to share and obtain data and analysis methods.

In this work, we present PyCellBase, a Python package to retrieve information from CellBase through its RESTful web services, which enables quick and easy access to heterogeneous biological information. No local database is required, reducing setup, administration, and maintenance costs. By implementing resource-specific clients and methods to fetch data from CellBase, we provide users with a straightforward way to access CellBase biological data with no need to know how its RESTful web services are implemented. Also, PyCellBase allows the integration of this information into custom Python scripts and pipelines for further processing and analysis.

PyCellBase can be easily installed from the Python Package Index (PyPI) (https://pypi.org/project/pycellbase/). It is a collaborative open-source project and its source code is conveniently distributed together with CellBase at GitHub (https://github.com/opencb/cellbase). Documentation and tutorials can be found at http://docs.opencb.org/display/cellbase. PyCellBase is released under Apache Software License, Version 2.0 and it is compatible with both Python 2 and 3.

## Availability and requirements

**Project name:** PyCellBase.

**Project home page:** https://pypi.org/project/pycellbase

**Operating system(s):** any supporting Python> = 2.7 (tested on Linux).

**Programming language:** Python.

**Other requirements:** requests> = 2.9.1, pyYAML> = 3.11.

**License:** Apache Software License, Version 2.0.

**Any restrictions to use by non-academics:** none.

## Additional file

Additional file 1: PyCellBase use case. Example of usage of the PyCellBase REST client library. (IPYNB 15 kb)

## Abbreviations

API: Application Programming Interface
gRPC: gRPC Remote Procedure Calls
HGVS: Human Genome Variation Society
JSON: JavaScript Object Notation
NHS: National Health System
PyPi: Python Package Index
REST: Representational State Transfer
TFBS: Transcription factor binding site
URL: Uniform Resource Locator
XREF: eXternal REFerence
